# Reconstruction of chronic radiation-induced ulcers in the chest wall using free and pedicle flaps

**DOI:** 10.3389/fsurg.2022.1010990

**Published:** 2022-11-08

**Authors:** Bo Zhou, Ying Long, Sha Li, Chunliu Lv, Dajiang Song, Yuanyuan Tang, Liang Yi, Zhenhua Luo, Gaoming Xiao, Zan Li, Xiao Zhou

**Affiliations:** ^1^ Department of Breast Oncoplastic Surgery, Hunan Cancer Hospital and the Afliated Cancer Hospital of Xiangya School of Medicine, Central South University, Changsha, China; ^2^ Translational Medicine Centre, Hunan Cancer Hospital and the Afliated Cancer Hospital of Xiangya School of Medicine, Central South University, Changsha, China; ^3^ Department of Thoracic Surgery, Hunan Cancer Hospital and the Afliated Cancer Hospital of Xiangya School of Medicine, Central South University, Changsha, China

**Keywords:** chest wall reconstruction, radiation-induced ulcers, 3Dprinting, pedical flap, free flap

## Abstract

**Background and purpose:**

Resection of radiation-induced ulcers often causes full-thickness defects of the chest wall. We retrospectively reviewed and evaluated 17 patients to explore a method of chest wall reconstruction.

**Materials and methods:**

A total of 17 breast cancer patients with radiation-induced ulcers were included. Various type of prostheses and flaps were used, results of clinic were evaluated.

**Results:**

Sixteen patients had full-thickness defects and one patient had only a soft tissue defect and underwent reconstruction with a pedicle latissimus dorsi (LD) myocutaneous flap. Among all 16 full-thickness defect cases, 15 patients underwent bony thoracic reconstruction using polymesh/3D-printed titanium plates or methyl methacrylate. For soft tissue reconstruction, 13 patients reconstruction using a free deep inferior epigastric perforator (DIEP) flap in combination with a contralateral transverse rectus abdominis myocutaneous (TRAM) flap, and 2 underwent pure free DIEP flap reconstruction. Among all the patients 15 healed with no complications, and 2 patients had delayed healing on the edges of the flaps.

**Conclusions:**

Distant pedicle or free flap can used for soft tissue defect coverage, for those severe patients with full-thickness defects and used prostheses, free deep inferior epigastric perforator flap in combination with a contralateral transverse rectus abdominis myocutaneous flap (TRAM + DIEP) would be an applicable choice.

## Introduction

Many breast cancer patients need radiotherapy after mastectomy. Radiotherapy is a double-edged sword: it can reduce the rate of local recurrence, but it may also have severe adverse effects on normal tissue. Radiation-induced chest wall ulcers are among the toughest complications after radiotherapy.

An ulcer that is induced by radiation therapy cannot heal by itself because radiation damage surrounding tissues, reduce the blood supply to the skin, and induce fibrosis, which impairs cellular repair. Studies have shown that radiation causes high concentrations of matrix metalloproteinase, which is hostile to cell replication (1). Moreover, lack of contraction that is caused by delayed myofibroblast function and repeated wound contamination are also factors for refractory radiation ulcers. Due to lack of oxygen and nutrition, skin grafts cannot be used to repair radiation-induced ulcers. The use of a local flap is also not recommended because tissue that surrounds the ulcer has been compromised by radiotherapy. Ribs and the sternum may also be involved in severely affected patients. The use of a well-vascularized distant flap is recommended for those patients. With free flaps, choosing a suitable recipient vessel is a substantial challenge, especially on the chest wall, which is impacted by radiotherapy.

In this study, we retrospectively examined data on patients who suffered from chest wall radiation ulcers. Free flaps and pedicle flaps were used in those patients, and the recipient vessels and clinical effects were analyzed.

## Materials and methods

### Ethics approval and participate enrollment

This study was approved by the institutional ethics committee of Hunan Cancer Hospital, and written informed consent was obtained from all patients. The clinical data of patients from August 2016 to June 2021 were reviewed. The demographic information and complications were analyzed.

All patients underwent biopsies when admitted to the ward to eliminate the possibility of cancer recurrence or radiation-induced sarcoma, none of them received reconstruction surgery before. Seventeen female patients were included in this report, all of whom were diagnosed with breast cancer after mastectomy and received radiotherapy to the chest. Their ages ranged from 43 to 72 (mean age: 57 years), the ulcer size in the chest ranged from 1.5 cm × 2.0 cm to 15 cm × 10 cm, and the ulcer duration ranged from 2 months to 60 months (mean duration: 12 months). Sixteen patients had full-thickness defects after extended resection of lesions. Because ribs or the sternum were involved, rib defects were reconstructed with methyl methacrylate and polymesh (PROLENE ETHICON, INC) in 9 patients and with 3D-printed titanium plates and polymeshin 6 patients. In 1 patient, a soft tissue defect of the chest was reconstructed with a latissimus dorsi myocutaneous flap. In 1 patient, a filleted arm flap was used due to complete loss of upper limb function. Free deep inferior epigastric perforator (DIEP) flaps or DIEP in combination with transverse rectus abdominis myocutaneous (TRAM) flaps were used in 15 patients.

### Preoperative preparations

The biopsy pathology results of all patients were confirmed to exclude recurrent cancer or radiation-related malignancy. All patients underwent chest wall MRI and CT scanning to define the involved borders of the soft tissue and skeletal thorax. Enhanced tomography angiography (CTA) of the inferior epigastric artery and internal thoracic artery was also completed to facilitate the development of surgical strategies. Most ulcer beds contaminated by many types of bacteria; hence, bacterial culture and drug sensitivity testing were completed before surgery. If the bony thorax was involved, the ribs or sternum removed; if the bony defect was larger than 5 cm in diameter or more than 3 ribs were removed, the bony thorax was reconstructed using various materials according to the defect and the financial situation of the patient. If we used a DIEP flap for soft tissue coverage, we also used a hand-held Doppler to confirm the location of the inferior epigastric artery perforator in the abdomen.

### Multidisciplinary approach

Plastic surgeons, thoracic surgeons, and orthopedists surgeons discussed each case the day before the operation. According to the results of the imaging examination, they decided the range of excision, designed the flap for defect reconstruction and assessed possible recipient vessels.

### Surgical technique

All patients underwent daily dressing changes for each ulcer bed to obtain a relatively clean wound surface. According to the plan that was established before the operation, the thoracic surgeon performed lesion excision, and the boundary of the excision extended beyond the ulcer because radiotherapy destroyed adjacent tissue. Generally, 2–3 cm of surrounding relatively healthy soft tissue should be removed to ensure better wound healing after the operation. When the ribs and sternum were involved, chronic osteomyelitis was usually observed, the unhealthy ribs or sternum were also removed, and patients developed full-thickness defects of the chest wall. If a defect was larger than 5 cm in diameter or included ≥3 ribs, skeletal thorax reconstruction was needed using methyl methacrylate or 3D-printed titanium plates and polymesh. In some patients, when the unhealthy ribs and part of the sternum were removed, the internal thoracic artery and vein were exposed, and the plastic surgeons assessed these vessels to decide if they could be used as recipient vessels. In some cases, the thoracic artery was destroyed by radioactive rays, and the plastic surgeons had to find other vessels, such as the lateral thoracic vessels, thoracoacromial vessels, branch of the thoracic dorsal vessels and subscapular blood vessels, transverse cervical vessels and superior thyroid vessels are alternative choices as recipient vessels. Usually, a team of plastic surgeons performed flap raising at the same time, and we often chose a free DIEP flap in combination with a contralateral TRAM flap as a reconstruction flap (It's a single flap, which has two blood vessel pedicels, one is superior epigastric artery, a pedicled myocutaneous source, and the other is inferior epigastric artery, a free pedicle source) if the defect was extensive. Thoracic surgeon did the skeletal thorax reconstruction. In order to repair the full thick defect of the chest wall, they sutured a layer of polypropylene knitted nonabsorbable mesh (PROLENE ETHICON, INC) to the surrounding tissue. Then, they fixed the 3D-printed titanium plate on the residual ribs if the defect is as large as mentioned above. Alternatively, making an artificial rib with methyl methacrylate would also be chosen when the defect didn’t involve the sternum.. When the thoracic surgeon finished the skeletal thorax reconstruction, the flap was transferred to the chest wall. The defect was repaired by a structure like “sandwich” include three layers: mesh-bony framework-flap. Finally, microsurgical end-to-end anastomosis was performed between the flap and the recipient vessels.

After the operation, the color of the flap, the capillary refill time, and the temperature of the flap were monitored. The survival of the flap, reoperation rate and complications of the donor site were also analyzed.

## Results

In all the patients except the patient who had only a soft tissue defect, full-thickness chest wall defects were observed, and rib defects were reconstructed with 3D printed titanium plates (6 patients) or methyl methacrylate (9 patients). In 1 patient, only polymesh was used to repair the skeletal thorax because the diameter of the defect was less than 5 cm.

To repair soft tissue defects, a filleted flap was used for 1 patient due to complete loss of upper limb function, and we had to remove her upper limb. A pedicle latissimus dorsi myocutaneous flap was used in 1 patient because the soft tissue defect was relatively small. For the remaining 15 patients, free DIEP in combination with contralateral TRAM flaps were used; among those cases, a bilateral DIEP flap was used for 1 patient because both sides of the internal thoracic artery had been ruined by lesions in the distal end. A DIEP flap in combination with a free TRAM flap was used in another patient. Due to weak perfusion of the superior epigastric artery, we had to cut off the rectus abdominis and anastomose the inferior epigastric artery. A pure free DIEP flap was used in 1 patient due to the relatively small defect. In 12 patients, free DIEP in combination with pedicle TRAM flaps were used. The size of each flap ranged from 15 cm × 6 cm to 40 cm × 15 cm. All free flaps need recipient vessels in the chest wall or neck. Among the 15 patients for whom free DIEP flaps were used, the thoracoacromial artery and vein were used as recipient vessels in 3 patients, the lateral thoracic vessels in 2 patients, and the superior thyroid artery and jugular vein or branches in 3 patients. Branches of the thoracodosal artery and vein were used in 2 patients. Subscapular vessels were used in 1 patient. The transverse cervical artery and vein were used in 1 patient. The proximal part of the internal thoracic artery and vein was used in 1 patient. The bilateral proximal part of the internal thoracic artery was used in 1 patient due to the presence of two blood vessel pedicels, and thoracic vessels and lateral thoracic vessels were used in 1 patient due to the presence of two sets of blood vessel pedicles.

All defects and donor sites were closed directly, and all flaps survived. Two patients had delayed wound healing on the margin of the flap, which healed with dressing changes for several days and required no additional surgical treatment. The patients were followed in the outpatient department for 2 to 58 months after the operation (average: 21.4 months). No ulcer recurrence was observed in any patient (Table 1). We've got relatively good clinic result by using different kinds of flap in this study, proper flap selection and recipient vessel choice is very important, all of these should be fully discussed before operation for different patients, and necessary modify may needed according to different situation during the operation. For those patients with large defect, DIEP flap in combination with a contralateral TRAM is a better choice due to its reliable blood perfusion and time saving procedure.

**Table 1:** Patients information and operation data.

Patient	Age	BMI	Ulcer size, cm^2^	Duration time, mo	Bony involved	Bony reconstruction	Type of flap	Size of flap, cm^2^	Recipient vessels	Follow- up, mo	Complications
1	43	25.4	4 × 3	36	2/3 ribs + PS	MM	DIEP	28 × 14	TA	58	No
2	58	23.7	5 × 10	60	2/3/4 ribs	MM	DIEP + TRAM	30 × 14	LTA	40	No
3	68	20.4	6 × 8	6	3/4/5/6ribs + S	Ti	DIEP + TRAM	30 × 12	TA	36	Delayed healing
4	56	25.1	4 × 4	12	3/4/5/6 ribs + PS	MM	DIEP + TRAM	30 × 13	TA	35	No
5	55	20.1	15 × 10	12	2/3/4 ribs	Ti	FF	28 × 15	no	33	Delayed healing
6	50	17.6	2.5 × 3.0	5	4/5/6 ribs + S	Ti	D-DIEP	28 × 12	D-ITA	27	No
7	62	23	3 × 4	36	2/3/4ribs + S	Ti	DIEP + TRAM	36 × 13	STA	24	No
8	59	24.6	6 × 8	30	2/3/4 ribs + S	Ti	DIEP + FTRAM	32 × 13	ITA + LTA	21	No
9	62	25.4	3.5 × 2.5	11	3/4/5 ribs + S	Ti	DIEP + TRAM	38 × 15	STA	22	No
10	57	26.4	1.5 × 2.0	5	3/4/5 ribs + S	MM	DIEP + TRAM	40 × 15	SA	22	No
11	63	19.5	2.5 × 3.0	12	4/5 ribs + PS	MM	DIEP + TRAM	32 × 15	TCA	11	No
12	53	28	6 × 6	36	3/4 ribs + PS	MM	DIEP + TRAM	34 × 13	LTA	11	No
13	39	28.8	6 × 5	2	2/3/4/5 ribs + PS	MM	DIEP + TRAM	34 × 13	ITA	9	No
14	46	26.3	4 × 3	12	No	no	LDM	15 × 6	no	7	No
15	71	28.7	5 × 4	5	3/4 ribs	no	DIEP + TRAM	35 × 17	TDA	3	No
16	57	20.7	2 × 1.5	24	2/3/4/5 ribs + PS	MM	DIEP + TRAM	35 × 12	TDA	3	No
17	72	28.7	6 × 6	24	2/3 ribs + PS	MM	DIEP + TRAM	36 × 14	STA	2	No

## Case reports

### Case 1

A 62-year-old female who underwent mastectomy and radiotherapy 22 years ago suffered from a chest wall ulcer for 36 months. She was treated by debridement, and the first rib, the cranial of the sternum and part of the clavicle were removed. Defects of the chest were reconstructed by titanium mesh and covered by a local flap in another hospital, but the wound failed to heal.

**Figure 1:**
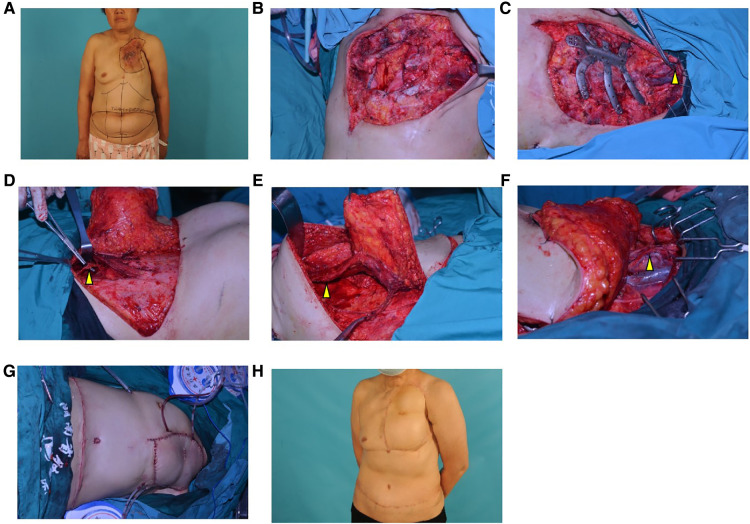
Surgical procedure and postoperative appearance of case 1 (**A**), A preoperative photo. We designed a DIEP flap in combination with a TRAM flap to cover the defect; (**B**), The lesion and involved ribs and sternum were removed, and a large full-thickness defect remained on the chest wall (25 cm × 14 cm); (**C**), A layer of polypropylene knitted nonabsorbable mesh was sutured to the surrounding tissue, and 3D-printed titanium plates were fixed to the residual ribs. The recipient vessel (the arrow shows the superior thyroid artery and the branch of the jugular vein) was prepared; (**D**), The DIEP flap was raised on the left side of the abdomen, and the arrow shows the inferior epigastric artery and vein; (**E**), The TRAM flap was raised on the right side of the abdomen. The arrow shows the muscle pedicle; (**F**), The flap was transferred to the chest wall, and microsurgical end-to-end anastomosis was performed between the flap and the recipient vessels. (The white arrow shows the superior thyroid artery and the yellow arrow shows the branch of the jugular vein.); (**G**), An immediate postoperative photo; (**H**), After 24 months of follow-up.

Outpatient biopsy showed no evidence of malignant changes. A chest wall CT scan revealed that the ulcer lesion involved 2–4 ribs and the sternum. A multidisciplinary discussion was conducted before the operation, and the excision and reconstruction methods were selected. A 3D-printed titanium plate was prepared according to the CT data. The range of excision was also identified under the guidance of the 3D-printed template. Wide excision of the lesion was carried out by thoracic surgeons. All unhealthy tissue was removed, including the involved 2–4 ribs and the sternum and pleura, which left a large full-thickness defect in the chest wall (25 cm × 14 cm). To repair this defect, we sutured a layer of polypropylene knitted nonabsorbable mesh (PROLENE ETHICON, INC) to the surrounding tissue, fixed the 3D-printed titanium plate on the residual ribs, and covered the titanium plate with a DIEP flap in combination with a TRAM flap. Because the internal thoracic artery had been ruined by the lesion and we failed to find any other recipient vessel in the chest wall or axilla, we had to find recipient vessels in the neck. Finally, we chose the superior thyroid artery and the branch of the jugular vein as recipient vessels. The donor site in the abdomen was closed directly, and the flap on the chest healed without any complications. This patient was followed for 24 months without recurrence of the ulcer (Figure 1).

## Case 2

**Figure 2:**
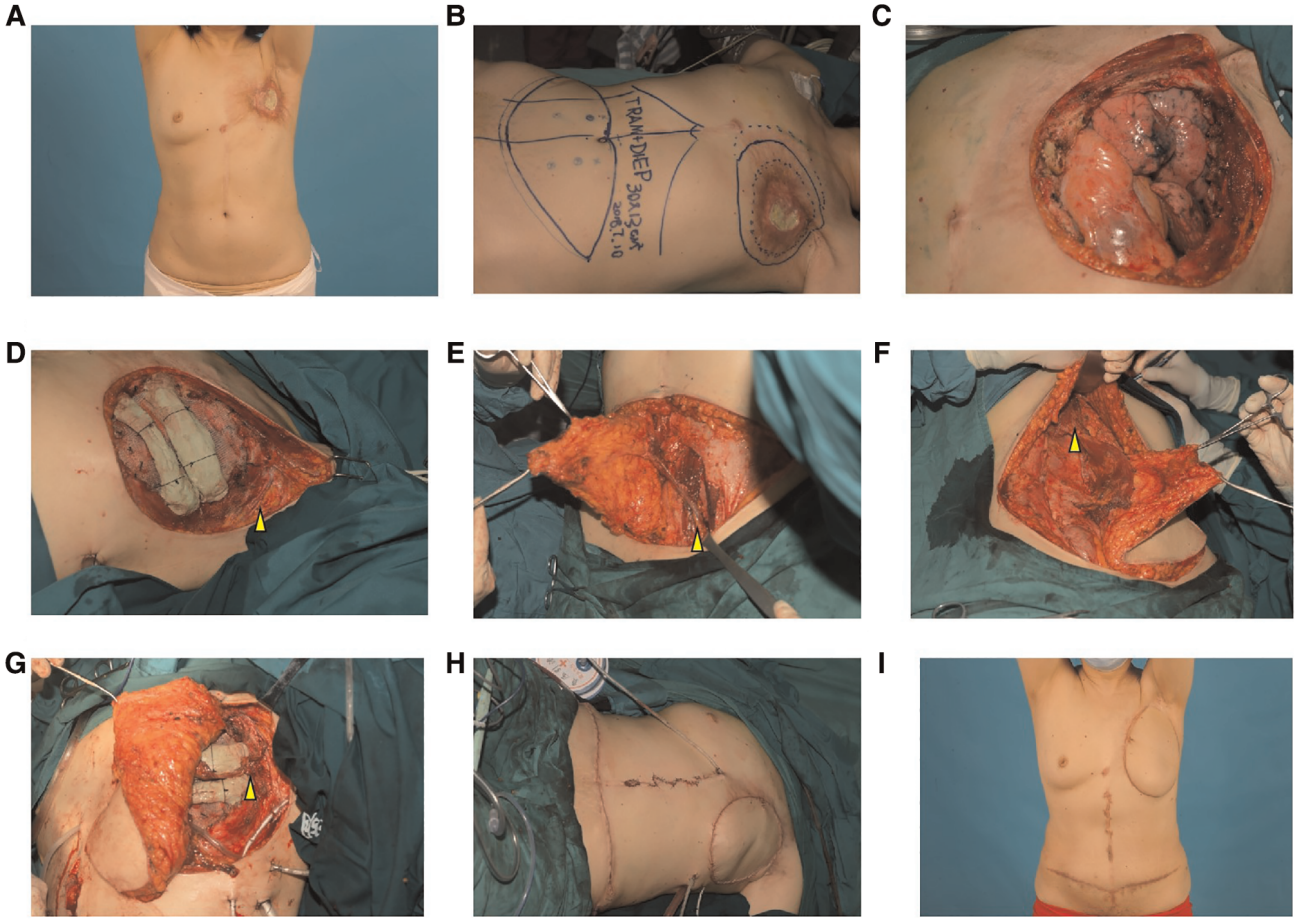
Surgical procedure and postoperative appearance of case 2 (**A**)**,** A preoperative photograph; (**B**), We designed a DIEP flap in combination with a TRAM flap to cover defects of the chest wall; (**C**), extensive excision of the lesion and the resulting huge full-thickness defect of the chest wall; (**D**), polypropylene knitted nonabsorbable mesh was sutured to the surrounding tissue, and two bars of methyl methacrylate were fixed on the residual rib above the polymesh. We dissected the thoracic acromial artery and vein as recipient vessels, as indicated by the arrows; (**E**), The DIEP flap was raised on the left side of the abdomen, and the arrow shows the inferior epigastric artery and vein; (**F**), The TRAM flap was raised on the right side of the abdomen, and the arrow shows the muscle pedicle; (**G**), The flap was transferred to the chest wall, and microsurgical end-to-end anastomosis was performed between the flap and the recipient vessels. (The arrow shows the thoracic acromial artery and vein); (**H**), Immediate postoperative photograph; (**I**), After 35 months of follow-up.

A 56-year-old female who underwent mastectomy and radiotherapy 16 years ago suffered from a radiotherapy-related ulcer on the chest wall for 12 months. Outpatient biopsy revealed no hint of malignancy. CT scans of the chest showed that 2–5 ribs and the left part of the sternum were involved. In a multidisciplinary discussion, we decided to extend the excision of the involved soft tissue and bones, reconstruct using polymesh and methyl methacrylate, and cover soft tissue defects using a DIEP flap in combination with a TRAM flap. The internal thoracic artery and vein were destroyed by the lesion; thus, we had to use the thoracic acromial artery and vein as recipient vessels. The donor site on the abdomen was closed directly, and the wound on the chest healed without any complications. This patient was followed for 35 months after surgery, without recurrence of the ulcer (Figure 2).

## Discussion

Many breast cancer patients need adjuvant radiation therapy, and radioactive rays not only kill possible residual tumor cells but also injure normal tissue around the radiation field.

Radiation may cause DNA damage and influence the proliferation function of cutaneous tissue, which is why a delayed radiation-induced ulcer may develop long after irradiation. The results of various studies show that the influence of radiation may continue for more than 20 years (2, 3). Radiotherapy-induced ulcers cannot heal by themselves, and they can last for months or years. Ulcers of this type fail to heal due to decreased angiogenesis and persistently high concentrations of matrix metalloproteinases, which create a hostile microenvironment for cell replication (4). In most patients with radiation ulcers on the chest wall, the lesions involve not only cutaneous tissue but also the bony thorax. Extensive resection of such lesions usually causes full-thickness defects, and reconstruction of those defects is always a major challenge to surgeons. Those operations also require tight cooperation between thoracic surgeons and plastic surgeons. Before surgery, multidisciplinary discussion is always necessary.

Generally, lesion resection and bony thoracic reconstruction are performed by thoracic surgeons. Extensive soft tissue resection is important because the tissue that surrounds an ulcer was usually in the radiotherapy field and destroyed by radioactive rays. Chronic ulcers that are sustained for a long time and contaminated by all kinds of bacteria often cause chronic osteomyelitis and osteonecrosis; hence, the involved ribs and sternum should also be removed (5, 6). For full-thickness defects, skeletal thorax stability should be re-established, and there are currently no consensus standards for chest wall reconstruction. However, most surgeons suggest that defects that are larger than 5 cm in diameter or involve more than 4 ribs should be reconstructed to avoid the risk of lung herniation and paradoxical motion of the chest wall (7). With the development of materials and technology, some surgeons advocate that nearly all chest wall defects should be reconstructed (8). The ideal prosthetic material for re-establishing chest wall stability should be malleable enough to conform to the shape of the chest wall, rigid enough to prevent paradoxical motion, radiolucent and biologically inert (9). In this study, we used 3D-printed titanium plates and methyl methacrylate to reconstruct the bony thorax, and they each have advantages and disadvantages. Titanium plates can be prepared before surgery from the data of the CT scan, and they are rigid enough and more suitable for the shape of the chest wall but more expensive. Methyl methacrylate is much cheaper, but it should be reshaped during surgery and is not permeable to fluids, which contributes to seroma formation and increases the risk of wound infection (10). Compared to titanium plates, it is also inferior in terms of mechanical properties.

Soft tissue defects are often reconstructed by plastic surgeons, and a defect that results from extended resection of a radiation-induced ulcer requires a well-vascularized flap, especially when prosthetic material has been implanted, such as a titanium plate or methyl methacrylate. A local flap may not be suitable for this scenario because the tissue that surrounds the ulcer has usually been impaired by radioactive rays. Distant pedicles or free flaps may be needed in most patients. In this study, DIEP flaps were used in 15 out of 17 patients (88%). When using free flaps, the selection of recipient vessels is very important. Some significant recipient vessels may also be destroyed by radioactive rays or ulcer lesions, such as internal thoracic arteries, which are the most frequently used vessels in breast or chest wall reconstruction surgery. In this study, in 3 out of 15 free flap patients (20%), the internal thoracic artery and vein were used as recipient vessels. Lateral thoracic vessels, thoracoacromial vessels, branches of thoracic dorsal vessels and subscapular blood vessels were also chosen as recipient vessels when the internal thoracic artery and vein were ruined. In some severely affected patients, we could not find recipient vessels on the chest wall or axilla, and vessels in the neck, such as transverse cervical vessels and superior thyroid artery, were selected as alternatives because those vessels were relatively far away from the radiation field.

In this study, in 12 out of 15 free flap patients (80%), a DIEP flap was used in combination with a contralateral pedicle TRAM flap as a soft tissue defect recovery flap. Because most patients develop a large defect after extensive resection, a pure single pedicle DIEP flap may not be large enough, and the perfusion at the edge of the flap is usually not satisfactory, which may dramatically influence the outcome of the operation. Patients may need several debridement surgeries after the operation to treat partial necrosis of the flap. In particular, prosthetic material implantation may cause prosthesis infections and exposure, thereby leading to failure of the reconstruction operation and high clinical cost. A free DIEP flap in combination with a contralateral pedicle TRAM flap can provide sufficient soft tissue coverage with satisfactory perfusion of blood; moreover, it requires only one site of vascular anastomosis, which renders it very suitable for patients with limited recipient vessels.

## Conclusions

Extensive resection of radiation-induced ulcers on the chest often causes full-thickness defects. For reconstruction of the bony thorax, 3D-printed titanium plates or methyl methacrylate can be used, and distant pedicles or free flaps can be used for soft tissue defect coverage. A free DIEP flap in combination with a contralateral pedicle TRAM flap is suitable for chest wall reconstruction, with a low rate of complications.

## Data Availability

The original contributions presented in the study are included in the article/Supplementary Material, further inquiries can be directed to the corresponding author/s.
